# Novel homozygous *CLN3* missense variant in isolated retinal dystrophy: A case report and electron microscopic findings

**DOI:** 10.1002/mgg3.1308

**Published:** 2020-05-22

**Authors:** Kei Mizobuchi, Takaaki Hayashi, Kazutoshi Yoshitake, Kaoru Fujinami, Toshiaki Tachibana, Kazushige Tsunoda, Takeshi Iwata, Tadashi Nakano

**Affiliations:** ^1^ Department of Ophthalmology The Jikei University School of Medicine Tokyo Japan; ^2^ Department of Ophthalmology Katsushika Medical Center The Jikei University School of Medicine Tokyo Japan; ^3^ Division of Molecular and Cellular Biology, National Institute of Sensory Organs National Hospital Organization Tokyo Medical Center Tokyo Japan; ^4^ Division of Vision Research, National Institute of Sensory Organs National Hospital Organization Tokyo Medical Center Tokyo Japan; ^5^ Core Research Facilities for Basic Science, Research Center for Medical Science The Jikei University School of Medicine Tokyo Japan

**Keywords:** electroretinography, lysosomal storage disease, retina, transmission electron microscopy, whole‐exome and genome sequencing

## Abstract

**Background:**

Biallelic *CLN3* gene variants have been found in either juvenile‐onset neuronal ceroid lipofuscinosis (JNCL) or isolated retinal dystrophy. It has been reported that most JNCL patients carry a common 1.02‐kb deletion variant homozygously. Clinical characteristics of patients with biallelic *CLN3* missense variants are not well elucidated.

**Methods:**

We described a 26‐year‐old Japanese male patient with isolated retinal dystrophy. Whole‐exome sequencing (WES) and transmission electron microscopy (TEM) were performed.

**Results:**

Whole‐exome sequencing identified a novel homozygous *CLN3* missense variant [c.482C>T; p.(Ser161Leu)]. Ophthalmoscopy revealed retinal degeneration and macular atrophy, and later attenuated retinal vessels. Severely reduced responses were observed in both rod and cone electroretinograms. In TEM of the patient's lymphocytes, fingerprint profiles, which are specific findings in *CLN3*‐associated JNCL, were observed in 16/624 (2.56%) lymphocytes of the patient, who has never exhibited neurological signs during the 13‐year follow‐up period.

**Conclusion:**

Our results indicated that this novel *CLN3* missense variant is associated with teenage‐onset isolated retinal dystrophy. This is the first report of any patient with *CLN3*‐associated disorder in the Japanese population. Although fingerprint profiles have never been reported in *CLN3*‐associated isolated retinal dystrophy, these profiles were observed, albeit infrequently, suggesting that frequency of the fingerprint profiles might depend on variant types.

## Graphical abstract

Biallelic *CLN3* gene variants have been found in either juvenile‐onset neuronal ceroid lipofuscinosis or isolated retinal dystrophy. We described clinical and genetic characteristics of a Japanese patient with teenage‐onset isolated retinal dystrophy in whom a novel homozygous *CLN3* missense variant was identified. This is the first report of a *CLN3*‐associated genetic disorder in the Japanese population.

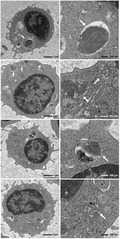

## 1 INTRODUCTION

Juvenile‐onset neuronal ceroid lipofuscinosis (JNCL, Batten disease, OMIM #204200) is a heterogeneous group of autosomal recessive neurodegenerative lysosomal storage disorders characterized by early‐onset retinal degeneration, epilepsy, and progressive psychomotor deterioration. The *CLN3* gene encodes a lysosomal/endosomal transmembrane protein of 438 amino acids and is ubiquitously expressed in human tissues (Mirza et al., 2019). Biallelic *CLN3* (OMIM *607042) variants have been associated with not only JNCL but also early‐ or late‐onset isolated retinal dystrophy without neurological signs (Chen et al., 2019; Ku et al., 2017; Lerner et al., 1995; Munroe et al., 1997; Wang et al., 2014). A previous study revealed that JNCL is associated with a common founder 1.02‐kb deletion variant of *CLN3*, demonstrating homozygous states in 74% (139/188 cases), compound heterozygous states in 22% (41/188 cases), and other biallelic variants in only 4% (8/188 cases) of patients (Munroe et al., 1997). To date, *CLN3*‐associated JNCL or isolated retinal dystrophy has never been reported in the Japanese population. Here, we described long‐term ophthalmological observation of a Japanese patient with isolated retinal dystrophy, identification of a novel homozygous *CLN3* variant, and characteristic electron microscopic findings.

## 2 MATERIALS AND METHODS

The Institutional Review Boards of The Jikei University (approval no. 24‐232 6997) and National Hospital Organization Tokyo Medical Center (R14‐050) approved the study protocol. The protocol adhered to the tenets of the Declaration of Helsinki, and informed consent was obtained from participants.

### 2.1 1 Clinical assessment

**FIGURE 1:**
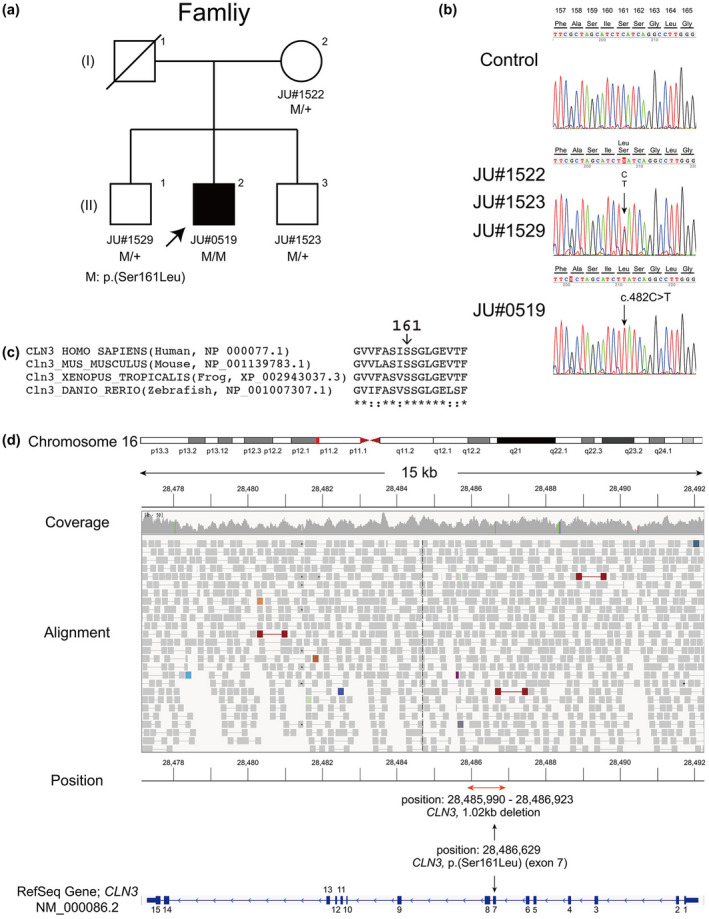
Pedigree chart, partial nucleotide sequences of *CLN3*, whole‐genome sequencing data. (a) The patient (male proband, JU#0519) and unaffected family (JIKEI‐010JIKEI) members (mother and two brothers) are shown. (b) A homozygous variant [c.482C>T; p.(Ser161Leu)] was detected in the patient, whereas all unaffected members (JU#1522, JU#1523, and JU#1529) carry the variant heterozygously. (c) Amino acid sequence alignments show that the Ser161 is well conserved among vertebrates. (d) The Integrative Genomics Viewer (IGV) visualization shows the [c.482C>T; p.(Ser161Leu)] variant at position 28,486,629 in GRCh38.p12, which is present within the region normally deleted in the common 1.02‐kb deletion. The coverage track on IGV reveals no decreased read depth around the p.(Ser161Leu) variant. Also, sequence read alignment data on IGV deny the possibility of presence of the 1.02‐kb deletion, although there are different three deletion regions with red lines, confirming that the patient carried the *CLN3* variant [p.(Ser161Leu)] homozygously

A 26‐year old‐male patient (II‐2, JU#0519, Figure 1a) from a Japanese family (JIKEI‐010JIKEI) was ophthalmologically assessed at The Jikei University Hospital. We performed a comprehensive ophthalmic examination, including decimal best corrected visual acuity (BCVA), slit‐lamp examination, funduscopy, fluorescein angiography, and fundus autofluorescence imaging (FAF) using an Optos 200Tx, Ultra‐Wide Field Retinal Imaging System (Optos), and spectral‐domain Cirrus optical coherence tomography (OCT; Carl Zeiss Meditec AG). Horizontal B‐scan OCT images through the fovea were obtained. Visual field testing using Goldmann perimetry (Haag Streit) was performed. Full‐field electroretinography (ERG) using a Ganzfeld dome and Neuropack 2 (Nihon Kohden) with corneal contact lens electrodes was recorded by an EOG‐ERG Ganzfeld stimulator (Electrophysiology system; LACE Elettronica) according to the protocol of the International Society for Clinical Electrophysiology of Vision (McCulloch et al., 2015). Details on the procedure and conditions have been previously reported (Katagiri et al., 2016; Kutsuma et al., 2019).

### 2.2 2 Molecular genetic study

Genomic DNA was extracted from venous blood samples of all participants (Figure 1a). Whole‐exome sequencing (WES) was performed as previously described (Katagiri et al., 2018). Whole‐genome sequencing (WGS) was also performed by Macrogen Japan. Briefly, paired‐end sequence libraries were constructed using the TruSeq DNA PCR Free Library Kit (Illumina). The libraries were subjected to 150‐bp paired‐end sequencing on the HiSeq X Ten system (Illumina). Reads from the fastq files were mapped/aligned to the human reference genome GRCh37/hg19 using Burrows‐Wheeler Aligner Software. Duplicate reads were removed using Picard Mark Duplicates. The mapped reads around insertions/deletions (Indels) were realigned by the Genome Analysis Toolkit (GATK) Version 3.0. Variant calling was performed using GATK HaplotypeCaller. The called single‐nucleotide variants (SNVs) and Indels were annotated using the SnpEff software 4.3. We focused on nonsynonymous variants and splice site variants, which are within 10 bp of exon–intron boundaries, and excluded synonymous and noncoding variants. We removed variants with allele frequency greater than 0.01 for recessive variants or 0.001 for dominant variants in any of the ethnic subgroups found in the following databases: 1,000 Genomes database (https://www.internationalgenome.org/), Genome Aggregation Database (https://gnomad.broadinstitute.org/), and Human Genetic Variation Database (http://www.hgvd.genome.med.kyoto‐u.ac.jp/).

In all available family members, we employed targeted next‐generation sequencing (NGS) and performed hybridization capture (xGen Predesigned Gene Capture Pools, Integrated DNA Technologies) experiments using *CLN3* exon‐specific oligonucleotide probes (Table S1), and amplicon sequencing using NextSeq 500 (Illumina), with an average depth of coverage of 700× for all targeted amplicons. The experiments were performed basically based on previously described methods (Fujiki et al., 2018) at Kazusa DNA Research Institute, Chiba, Japan.

A potential pathogenic variant in the *CLN3* gene was confirmed by Sanger sequencing for cosegregation in all available family members. The following primer set for exon 7 of the *CLN3* gene was used: forward primer 5′‐GTCTGATAACTGGGTGGATG‐3′ and reverse primer 5′‐AAGGAGAACACAGGAACATTC‐3′. The region around the identified *CLN3* variant was visualized using Integrative Genomics Viewer (IGV) browsers (Robinson et al., 2011). We used the transcript sequence (NM_000086.2) of the *CLN3* gene, and human CLN3 (NP_000077), mouse Cln3 (NP_001139783.1), frog Cln3 (XP_002943037.3), and zebrafish Cln3 (NP_001007307.1) protein sequences for alignment. Multiple amino acid sequence alignments were performed using Clustal Omega (https://www.ebi.ac.uk/Tools/msa/clustalo/).

### 2.3 3 Transmission electron microscopy analysis

Sample preparation from the patient's peripheral lymphocytes for transmission electron microscopy (TEM) was performed based on previously described methods (Hayashi et al., 2020; Kuniyoshi et al., 2020). Finally, ultrathin sections of about 70 nm were stained with uranyl acetate and lead citrate and observed on a JEM1400 Plus electron microscope (JEOL) at 100 kV.

## 3 RESULTS

### 3.1 1 Case presentation

**FIGURE 2:**
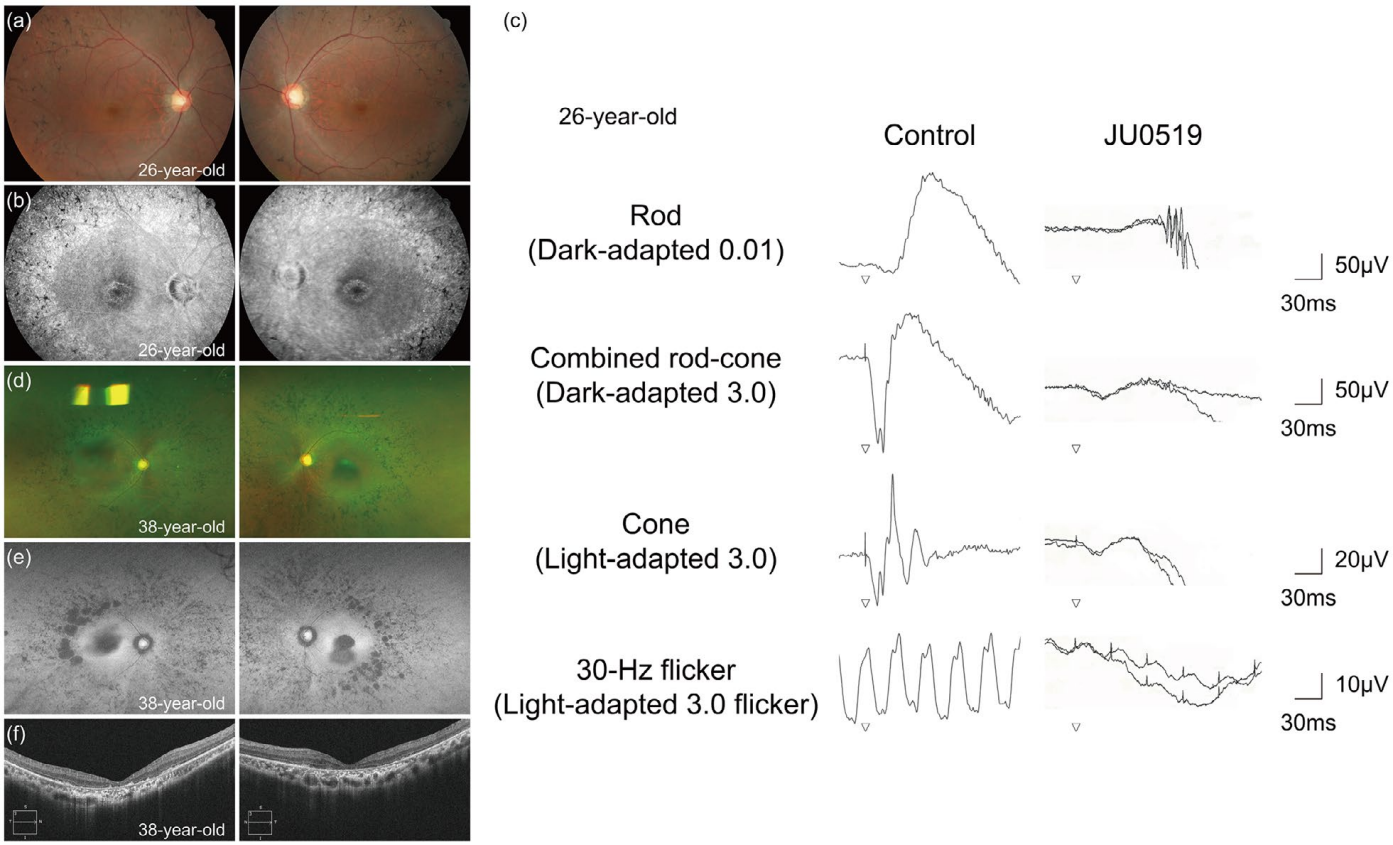
Multimodal retinal images and full‐field electroretinograms during the patient's clinical course. Fundus photographs (a), late‐phase fluorescein angiography images (b), and electroretinograms (c) taken at 26 years of age. Ultrawide‐field fundus photographs (d) and fundus autofluorescence images (e) taken at 38 years of age. Optical coherence tomography (f) shows disruption of all outer retinal layers at the center of the maculae. Left column: images of the right eye, right column: images of the left eye in (a), (b), (d)–(f)

**FIGURE 3:**
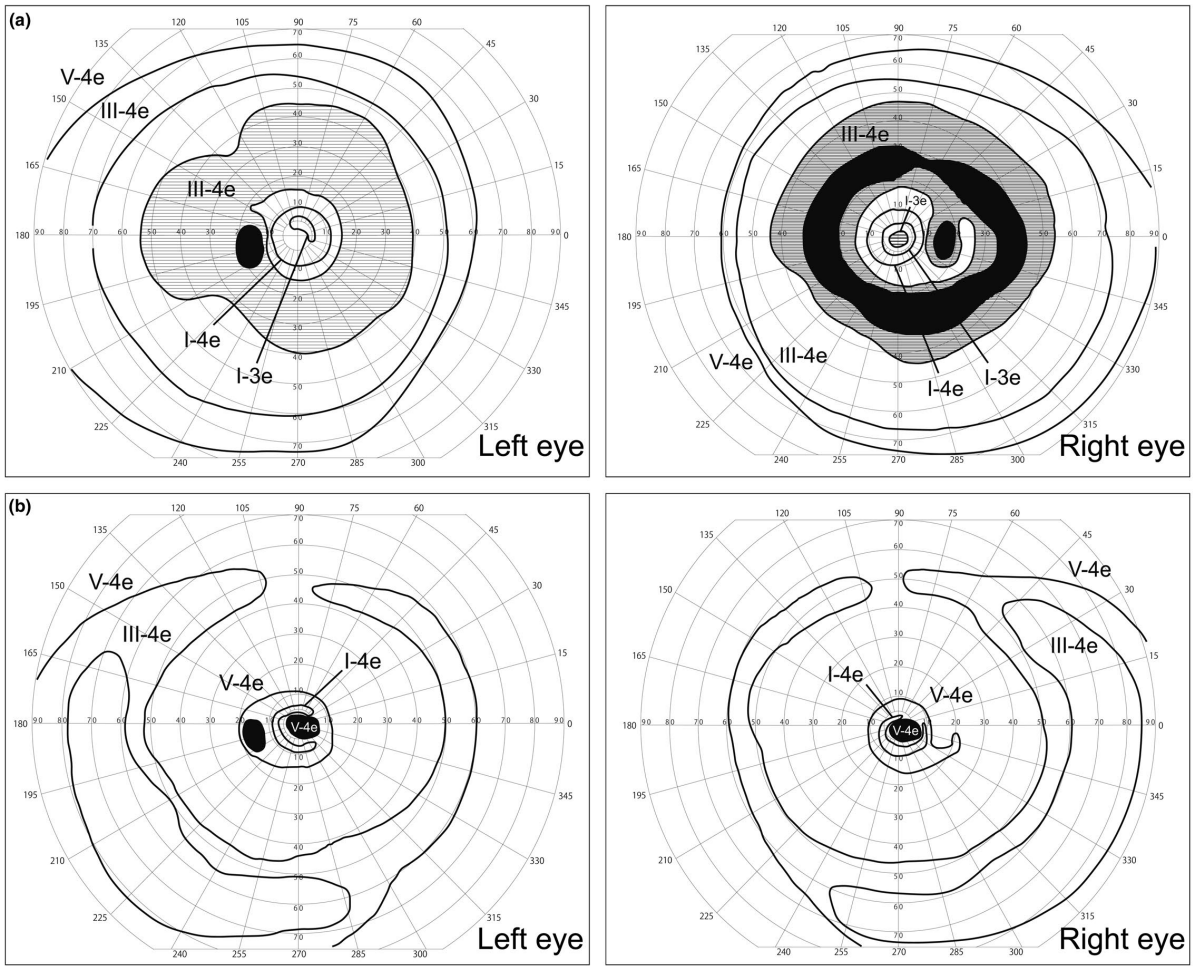
Visual fields with Goldmann perimetry. (a) At 26 years of age, scotoma patterns with decreased central sensitivities are observed in both eyes. (b) At 38 years of age, visual fields were progressively constricted in both eyes

The 26‐year‐old patient was referred to The Jikei University hospital for the assessment of poor visual acuity. He manifested night blindness in his early teens. His parents, who were nonconsanguineous and unaffected, were from the same prefecture in Japan. His decimal BCVA was 0.7 (Snellen equivalent 20/28, spherical − 4.50 diopter) in the right eye and 0.2 (Snellen equivalent 20/100, spherical − 4.25 D) in the left eye. No remarkable finding was observed in the anterior segments or media in both eyes (BE). Fundus photographs showed retinal degeneration with pigmentation and mild macular involvement in BE (Figure 2a). Late‐phase fluorescein angiography 10 min after injection showed hyperfluorescent patterns due to transmission defects in the maculae and outside vascular arcades in BE (Figure 2b), indicating degenerative changes in the retina and retinal pigment epithelium. GP showed ring scotoma patterns with decreased central sensitivities in BE (Figure 3a). Full‐field ERG findings revealed severely decreased responses in rod (dark‐adapted [DA] 0.01) ERG, in combined/rod‐plus‐cone (DA 3.0) ERG, cone (light‐adapted 3.0) ERG, and 30‐Hz flicker ERG (Figure 2c). Follow‐up observation revealed progressive deterioration of visual function. At 38 years of age, BCVA was 0.2 (Snellen equivalent 20/100) in BE. GP revealed that the visual fields were progressively constricted in BE (Figure 3b). Ultrawide‐field fundus photographs and fundus autofluorescence showed severe retinal degeneration with attenuated retinal vessels in BE (Figure 2d,e). OCT showed loss of the central macular outer layers in BE (Figure 2f). During the 13‐year follow‐up period, the patient has never exhibited neurological signs seen in protracted JNCL (Munroe et al., 1997). In protracted JNCL patients, the first progressive loss of vision occurs, with delayed neurologic onset, usually in the third or fourth decade.

### 3.2 2 Molecular genetic findings

After filtering WES data, 11 variants were investigated based on the patient's phenotype and inheritance pattern. Eventually, we identified a novel homozygous variant [c.482C>T; p.(Ser161Leu) in exon 7 (GRCh38chr16: 28486629)] of the *CLN3* gene (Figure 1b). The heterozygous *CLN3* variant was found in his unaffected mother and brothers (Figure 1a,b). Amino acid sequence alignments showed that the Ser161, located on the second luminal loop (Mirza et al., 2019), is well conserved among vertebrates (Figure 1c), suggesting functional importance for the residue. The variant was not found in the Genome Aggregation Database (https://gnomad.broadinstitute.org/) or the Human Gene Mutation Database (http://www.hgmd.cf.ac.uk/ac/index.php). According to the American College of Medical Genetics guideline (Richards et al., 2015) using the VarSome program (https://varsome.com/), p.(Ser161Leu) was classified as likely pathogenic based on the following lines: PM1 (hot spot of length 61 base‐pairs has 5 non‐VUS coding variants (5 pathogenic and 0 benign), pathogenicity = 100.0%), PM2 (the variant was not found in GnomAD exomes), PP2 (24 of 41 missense variants in *CLN3* were pathogenic), and PP3 (pathogenic computational verdict because nine pathogenic predictions from DANN, DEOGEN2, EIGEN, FATHMM‐MKL, M‐CAP, MVP, MutationAssessor, MutationTaster, and SIFT vs. two benign predictions from PrimateAI and REVEL). Unexpectedly, the p.(Ser161Leu) variant was present within the region normally deleted in the common 1.02‐kb deletion. A question was raised whether his deceased father might have carried the common 1.02‐kb deletion heterozygously. To resolve this issue, we performed WGS and targeted NGS to exclude presence of the heterozygous 1.02‐kb deletion in the patient. The coverage track on IGV revealed no decreased read depth around the p.(Ser161Leu) variant (Figure 1d), indicating no evidence of the heterozygous 1.02‐kb deletion. Sequence read alignment data on IGV also denied the possibility of presence of the 1.02‐kb deletion (Figure 1d). Homozygosity mapping using WGS showed a high rate of homozygosity (>80%) of single nucleotide variants around the region including the p.(Ser161Leu) variant (Figure S1). In addition, targeted NGS showed to be negative for any copy number variation including heterozygous deletion in each *CLN3* exon in the patient, as well as other family members and a control (Figure S2). These results confirmed that the patient carried the *CLN3* variant [p.(Ser161Leu)] homozygously.

### 3.3 3 Transmission electron microscopy

**FIGURE 4:**
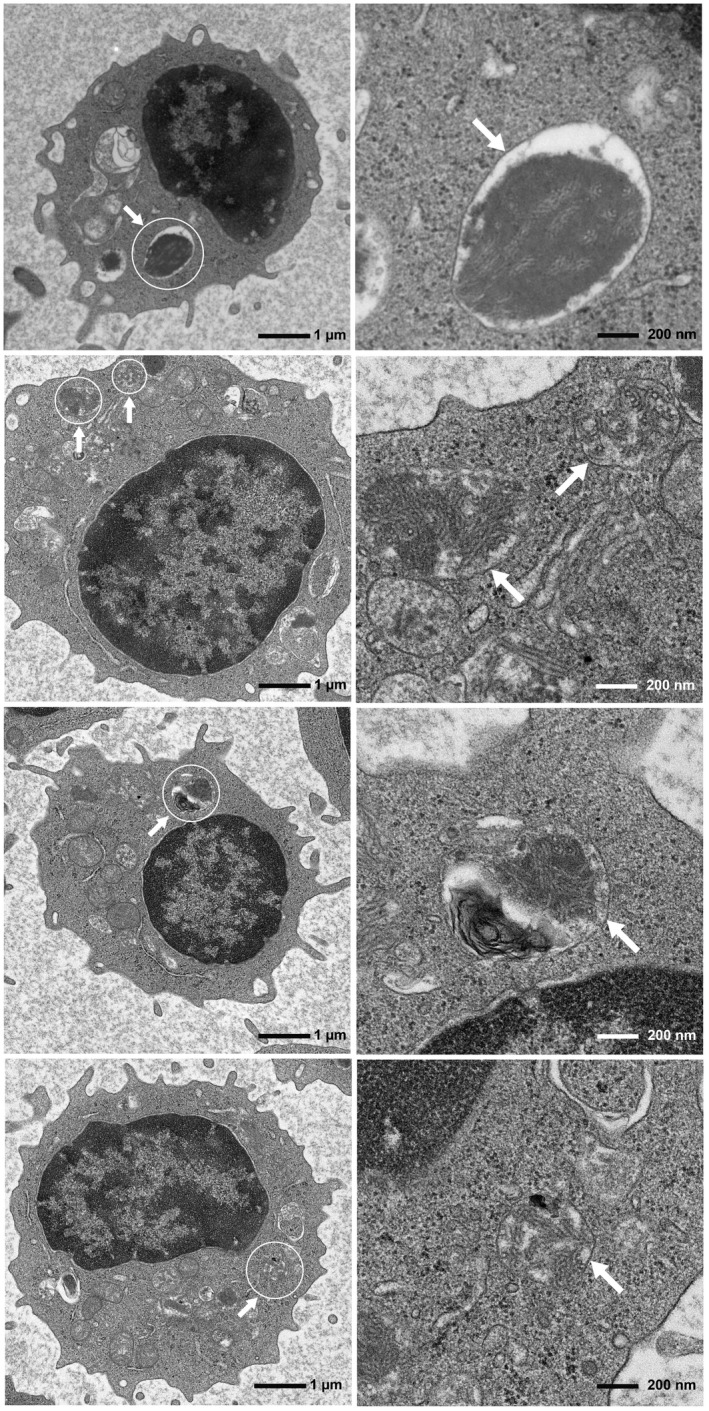
Transmission electron microscopic findings of four different lymphocytes. Images on the right panel are magnified images of circled areas in images on the left panel. Fingerprint profiles in each lysosome surrounded by a limiting membrane are present and depicted by arrows

Transmission electron microscopy was next used to evaluate the patient's lymphocytes. Generally, each lysosome is surrounded by a limiting membrane and contains various electron‐dense materials. Fingerprint profiles, which indicate lysosomal storage material, were observed in the patient (Figure 4). The fingerprint profiles, which are specific findings seen in *CLN3*‐associated JNCL, were observed in 16/624 lymphocytes (2.56%) analyzed.

## 4 DISCUSSION

In this study, we reported a patient with onset of night blindness in his teens and decreased visual acuity that progressively deteriorated over 13 years of follow‐up, who was found to carry a novel homozygous variant in the *CLN3* gene.

To date, 38 *CLN3* variants, which are associated with JNCL or isolated retinal dystrophy, have been reported (Chen et al., 2019; Kousi, Lehesjoki, & Mole, 2012; Ku et al., 2017; Lerner et al., 1995; Munroe et al., 1997; Sarpong et al., 2009; Wang et al., 2014; Wisniewski et al., 1998). As for genotype–phenotype correlations, the homozygous 1.02‐kb deletion has been found only in the most severe *CLN3*‐associated phenotype, JNCL (Lerner et al., 1995; Munroe et al., 1997). In contrast, to date, 16 patients with biallelic *CLN3* variants and isolated retinal dystrophy have been reported (Chen et al., 2019; Ku et al., 2017; Wang et al., 2014). The genetic details of these 16 patients are as follows (Table 1): seven patients (43.8%, Cases 2, 3, 5, 6, 7, 8, 9) had the 1.02‐kb deletion and missense or splice site variants, three patients (18.8%, Cases 12, 13, 14) had nonsense and missense variants, one patient (6.2%, Case 1) had splice site and missense variants, and five patients (31.2%, Cases 4, 11, 15, 16, 17) had biallelic missense variants. Only 11 of these 16 patients were ophthalmologically described in detail previously (Chen et al., 2019; Ku et al., 2017), which were summarized in Table S2. Among the 11 patients, 2 patients (Cases 16 and 17) carried a homozygous missense variant [p.(Arg405Trp)] and exhibited a milder phenotype (late‐onset isolated retinal dystrophy), including onset in their 30s, retinal degeneration without macular atrophy, preserved foveal ellipsoid zone in OCT, and no central scotoma (Ku et al., 2017), compared with our patient, who exhibited onset in his teens, progressive macular atrophy and central scotoma in GP, and severely decreased ERG responses (Figure 2c). Fingerprint profiles in TEM, which indicate lysosomal material storage, are considered a specific finding of JNCL (Santavuori, 1988). Moreover, previous studies have revealed that fingerprint profiles were not observed in three patients with isolated retinal dystrophy (Ku et al., 2017). However, our patient exhibited fingerprint profiles in lymphocytes, albeit infrequently (2.56% of analyzed lymphocytes) (Figure 4), which might explain why the patient did not manifest neurological signs. In the previous study (Ku et al., 2017), if more lymphocytes had been examined by TEM, the fingerprint profiles might have been seen in the three patients. It is speculated that low frequency of the fingerprint profiles may be associated with isolated retinal dystrophy without neurological signs. In *CLN3*‐associated isolated retinal dystrophy, further TEM analysis is needed for elucidating frequency of fingerprint profiles. The above‐mentioned consequences suggest that the frequency of fingerprint profiles might be positively correlated with disease severity.

**Table 1:** Biallelic *CLN3* variants of previously reported patients with isolated retinal dystrophy

Case #	Patient ID in original paper	*CLN3* variants		References
		Allele 1	Allele 2	
1	2044	c.125+1G>C (intron 2)	p.(Arg405Trp) (exon 15)	Wang et al. (2014)
2	Proband	p.(Ala59Thr) (exon 3)	1.02‐kb del (exon 7‐8)	Chen et al. (2019)
3	SK3	c.375‐3C>G (Intron 5)	1.02‐kb del (exon 7‐8)	Ku et al. (2017)
4	SRF41	p.(Ser131Arg) (exon 6)	p.(Glu295Lys) (exon 11)	Wang et al. (2014)
5	CEI1,	c.461‐3C>G (Intron 6)	1.02‐kb del (exon 7‐8)	Ku et al. (2017)
6	MEH3	1.02‐kb del (exon 7‐8)	c.837+5G>A (Intron 10)	Ku et al. (2017)
7	MEH5	1.02‐kb del (exon 7‐8)	p.(Ile285Val) (exon 11)	Ku et al. (2017)
8	MEH2	1.02‐kb del (exon 7‐8)	p.(Val330Ile) (exon 13)	Ku et al. (2017)
9	MEH6,	1.02‐kb del (exon 7‐8)	p.(Arg405Trp) (exon 15)	Ku et al. (2017)
10	JU#0519	p.(Ser161Leu) (exon 7)	p.(Ser161Leu) (exon 7)	This study
11	2691	p.(Gly189Arg) (exon 8)	p.(Gly189Arg) (exon 8)	Wang et al. (2014)
12	2055	p.(Val290Leu) (exon 11)	p.Tyr322* (exon 13)	Wang et al. (2014)
13	SK1	p.Glu295* (exon 11)	p.(Leu306His) (exon 12)	Ku et al. (2017)
14	SK2	p.Glu295* (exon 11)	p.(Leu306His) (exon 12)	Ku et al. (2017)
15	348	p.(Arg405Trp) (exon 15)	p.(Arg405Trp) (exon 15)	Wang et al. (2014)
16	MEH1	p.(Arg405Trp) (exon 15)	p.(Arg405Trp) (exon 15)	Ku et al. (2017)
17	MEH4	p.(Arg405Trp) (exon 15)	p.(Arg405Trp) (exon 15)	Ku et al. (2017)

As for the phenotype, although our patient did not exhibit any neurologic signs during follow‐up, two scenarios are considered in future. One possibility is that the patient has lifelong isolated retinal dystrophy, the other possibility is that the patient would present a protracted phenotype, manifesting first retinal dystrophy long before the manifestations of NCL become apparent.

In conclusions, we described clinical and genetic characteristics of a Japanese patient with teenage‐onset isolated retinal dystrophy in whom a novel homozygous *CLN3* missense variant was identified. This is the first report of a *CLN3*‐associated genetic disorder in the Japanese population. TEM findings of the patient's lymphocytes revealed characteristic fingerprint profiles, albeit infrequently. In consideration of genotype–phenotype correlations, severities of *CLN3*‐associated phenotypes might depend on variant types.

## CONFLICT OF INTEREST

The authors declare no competing financial interest.

## AUTHOR CONTRIBUTIONS

KM, TH, KY, KF, TT, KT, TI, and TN contributed to the conception and design of this research. KM and KY performed molecular genetic analysis. TT performed transmission electron microscopy. TH contributed to the clinical characterization of the patient. KM wrote the draft of this manuscript. All authors contributed to manuscript revision and approved the submitted final version.

## Supporting information

Fig S1

Fig S2

Table S1

Table S2

## Data Availability

The data that support the findings of this work are available from the corresponding author upon reasonable request.
