# Supplementary figures and images for: Novel homozygous CLN3 missense variant in isolated retinal dystrophy: A case report and electron microscopic findings
Source: Mol Genet Genomic Med. 2020 May 22;8(8):e1308. doi: 10.1002/mgg3.1308 (PMC7434607; doi:10.1002/mgg3.1308)

*CLN3*, p.(Ser161Leu)  
position: 28,486,629

Rate of homozygosity (%)

100  
75  
50  
25  
0

0

25

50

75

Position in chromosome 16 (Mega base pairs)

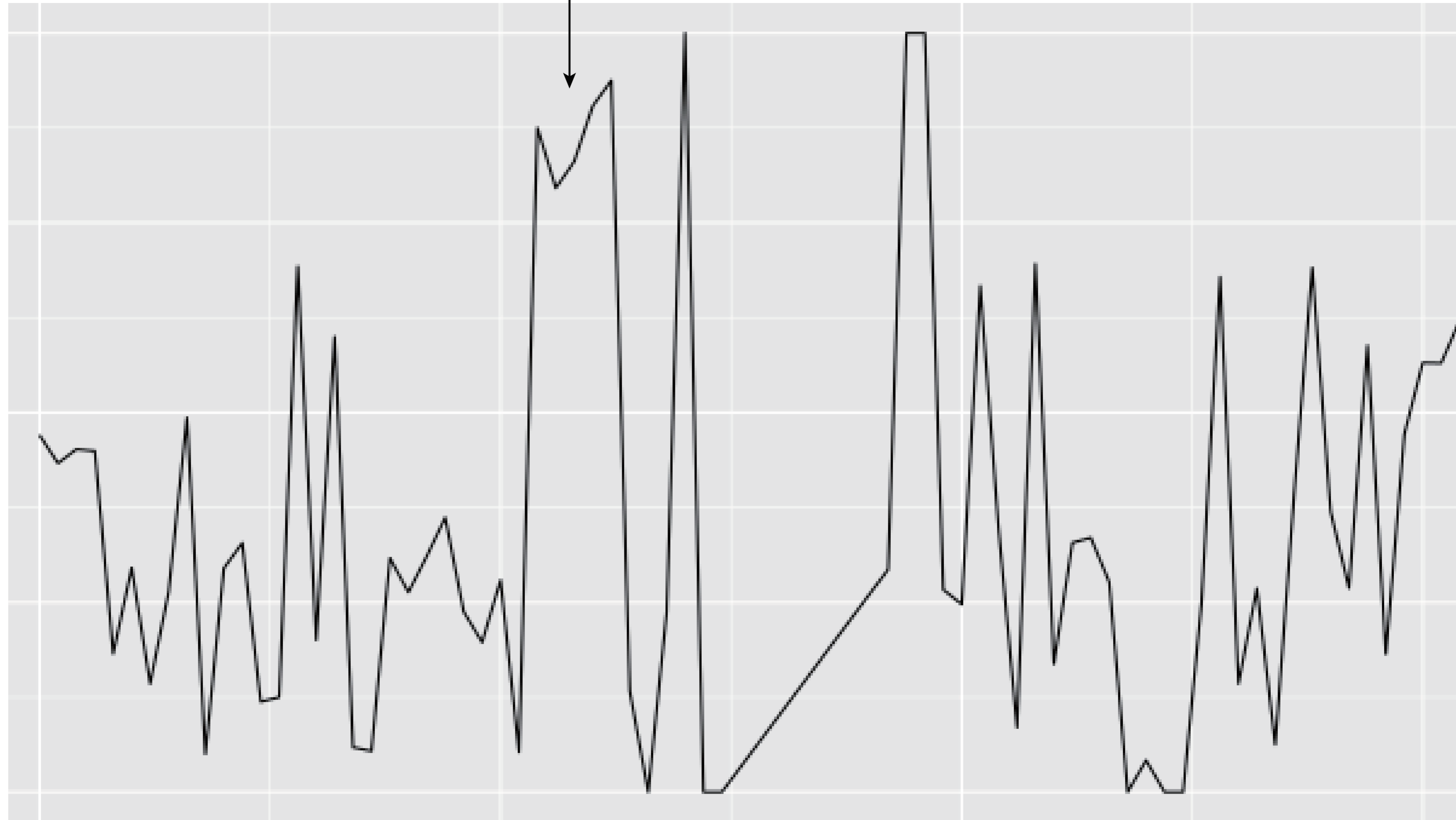

Supplement: Supplementary file 1 — Fig S1 [file MGG3-8-e1308-s001.pdf]

Patient (II-2)  
Mother (I-2)  
Brother (I-3)  
Brother (I-1)  
Control

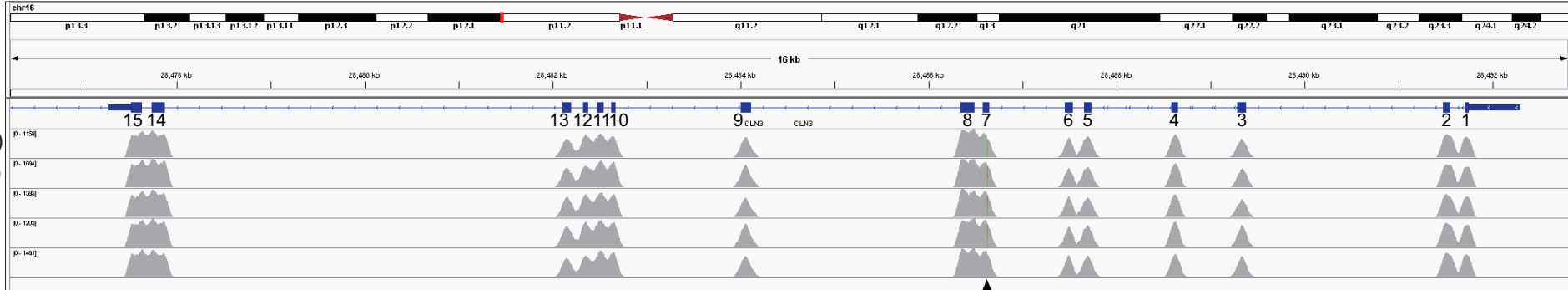

Supplement: Supplementary file 2 — Fig S2 [file MGG3-8-e1308-s002.pdf]
